# Supplementary material for: The Additional 15 nt of 5′ UTR in a Novel Recombinant Isolate of Chilli Veinal Mottle Virus in Solanum nigrum L. Is Crucial for Infection
Source: Viruses. 2023 Jun 23;15(7):1428. doi: 10.3390/v15071428 (PMC10384581; doi:10.3390/v15071428)
Supplement: Supplementary file 1 [file viruses-15-01428-s001.zip › Table S1.pdf]

Table S1. Primers used in this study

Objective	name	Sequence (5′-3′)
Full-genome sequence ChiVMV-YunN/Yuxi	ChiVMV-2L-1 f	AAAAATAAAACAACCAATACAAACATAC
	ChiVMV-2L-1 r	CAGGAACAGCTGCGAAGTAG
	ChiVMV-2L-2 f	GTTCCACAAAATTTGTACAGGAG
	ChiVMV-2L-2 r	AACGCCAACTATTGAACAGTTTAC
Virus detection	Det- ChiVMV f	GGAGAGAGTGTTGATGCTG
	Det- ChiVMV r	TTGCGCTTCTCAATGTACGC
15nt detection	UTR f	AAAAATAAAACAACCAATAC
	UTR r	TACTCTTCCGCACTCTTCG
	UTRd15 f	AATACAAACATACAGAAAAA
RACE PCR	M4	GTTTTCCCAGTCACGAC
	M4T	GTTTTCCCAGTCACGAC(T)15
	ZHM1	PO4-CTCTTCCCCTCCCTCCTC-NH2
	ZHM2	GAGGAGGGAGGGGAAGAG
	3'-RACE-F	TCAGTCGGGAGAGAGTGTTG
	5'-RACE-ChiVMV-YunN-R	ACCGGGCTAATTCTAGTAAAC
	5'-RACE-R	CAAGGATCCATATCTACCTCTG
Colony PCR-p ChiVMV-YunN/Yuxi	pCB-ChiVMV-YunN/Yuxi-1 f	CGAACAAACTCAAGCATTCAAG
	pCB-ChiVMV-YunN/Yuxi-1 r	CTTGATATCTGGATCCATGTC
	pCB-ChiVMV-YunN/Yuxi-2 f	GACATGGATCCAGATATCAAG
	pCB-ChiVMV-YunN/Yuxi-2 r	TGGAGATGCCATGCCGACCCAACGCCAACTATTGAACAGTTTAC
	Vec-pCB301 f	GGGTCGGCATGGCATCTCCA

	Vec-pCB301 r	CTTGAATGCTTGAGTTTGTTCGTTTTTCTGTATGTTTGTATTGGTTGTTTTATTTTTCCTCTCCAAATGAAATGAACTT
Colony pCR-pChiVMV-GFP	Vec-pChiVMV-GFP f	TCGGGAGAGAGTGTTGATGC
	Vec- pChiVMV-GFP r	TGCTCCAATACTCTAAGGCTG
	pChiVMV-GFP-1 f	CAGCCTTAGAGTATTGGAGCA
	pChiVMV-GFP-1 r	CTGATGGGTGACTTCTCCAC
	pChiVMV-GFP-2 f	GTGGAGAAGTCACCCATCAGtcgGGAATGGCAAGTAAAGGAGAAGAAC
	pChiVMV-GFP-2 r	GCATCAACACTCTCTCCCGACTGATGGGTGACTTCTCCACCCTCTTTGTATAGTTCATCCATGCCA
Colony pCR-pChiVMV$_{d15}$-GFP	Vec-pChiVMVd15-GFP f	TCGGGAGAGAGTGTTGATGC
	Vec-pChiVMVd15-GFP r	cctctccaaatgaaatgaactt
	pChiVMVd15-GFP-1 f	aagttcatttcatttggagaggAATACAAACATACAGAAAAACGAAC
	pChiVMVd15-GFP-1 r	TGCTCCAATACTCTAAGGCTG
	pChiVMVd15-GFP-2 f	CAGCCTTAGAGTATTGGAGCA
	pChiVMVd15-GFP-2 r	GCATCAACACTCTCTCCCGACTGATGGGTGACTTCTCCACCCTCTTTGTATAGTTCATCCATGCCA
Colony pCR-pChiVMV$_{MutC}$-GFP	pChiVMV-MutC-1 f	aagttcatttcatttggagaggCCCCCTCCCCCCCCC AATACAAACATACAGAAAAACGAAC
	pChiVMV-MutC-1 r	CTTCTGATGGCCTCTTACTTGC
	pChiVMV-MutC-2 f	GCAAGTAAGAGGCCATCAGAAG
	pChiVMV-MutC-2 r	CATACAACCACTTCTCGCTTGC
	pChiVMV-MutC-3 f	GCAAGCGAGAAGTGGTTGTATG
Colony pCR-pChiVMV$_{delete1A}$-GFP	pChiVMV-Del1A-1 f	aagttcatttcatttggagagg AAAATAAAACAACCAATACAAACATACAGA
Colony pCR-pChiVMV$_{Add1A}$-GFP	pChiVMV-Add1A-1 f	aagttcatttcatttggagagg AAAAAATAAAACAACCAATACAAACATACAGA

	ChiVMV-CP f	GGAGAGAGTGTTGATGCTG
	ChiVMV-CP r	TTGCGCTTCTCAATGTACGC
	GFP f	ATGGCAAGTAAAGGAGAAGAAC
	GFP r	TTTGTATAGTTCATCCATGCCAT
	Nb-UBC f	TTTCGGTCCTGATGATACTCCC
	Nb-UBC r	CACAGAGCAAAGACTGGATTGA

a. The Virus detection primers were used to verify the virus sequences.

b. The RACE PCR primers were used to obtain the 5’ and 3’ terminal sequences of ChiVMV-YunN/Yuxi.

c. The Full-genome sequence primers were used to obtain the complete genome sequence.

d. The Colony PCR primers were used to generate infectious clones pChiVMV, pChiVMV-GFP and $pChiVMV_{d15}$-GFP.
