# Supplementary material for: The Additional 15 nt of 5′ UTR in a Novel Recombinant Isolate of Chilli Veinal Mottle Virus in Solanum nigrum L. Is Crucial for Infection
Source: Viruses. 2023 Jun 23;15(7):1428. doi: 10.3390/v15071428 (PMC10384581; doi:10.3390/v15071428)
Supplement: Supplementary file 1 [file viruses-15-01428-s001.zip › Table S2.pdf]

Table S2. Accession numbers of potyviruses used for phylogenetic analysis

Abbreviations	Accession[a]	Country
ChiVMV-PK	MN207122.1	Pakistan
ChiVMV-Jal	GU170807.1	India
ChiVMV-War	GU170808.1	India
ChiVMV-Ta	MN508959.1	India
ChiVMV-Uravakonda	MN508960.1	India
ChiVMV-TaiW	OK181760.1	China
ChiVMV-KP	AM909717.1	Korea
ChiVMV-Ca	AJ972878.1	Korea
ChiVMV-HuN	LN832362.1	China
ChiVMV-GD	KU987835.1	China
ChiVMV-HuN/Ca	KR296797.1	China
ChiVMV-SiC/Wenchang	GQ981316.1	China
ChiVMV-GX	MT782116.1	China
ChiVMV-IN	AJ237843.3	India
ChiVMV-SiC/Yp8	KC711055.1	China
ChiVMV-SiC/Pp4	KC711056.1	China
ChiVMV-SiC/Luzhou	MK405594.1	China
ChiVMV-YunN/Dehong	MT787292.1	China
ChiVMV-YunN/Ca	MT974520.1	China
ChiVMV-YunN/Tobacco	JX088636.1	China
ChiVMV-YunN/Yuxi	OP404087*	China

* Sequence determined in this study.
