# Supplementary material for: The Additional 15 nt of 5′ UTR in a Novel Recombinant Isolate of Chilli Veinal Mottle Virus in Solanum nigrum L. Is Crucial for Infection
Source: Viruses. 2023 Jun 23;15(7):1428. doi: 10.3390/v15071428 (PMC10384581; doi:10.3390/v15071428)
Supplement: Supplementary file 1 [file viruses-15-01428-s001.zip › Table S3.pdf]

Table S3. Summary of possible recombination events among 26 genome sequences of ChiVMV isolates used by RDP5

Event Number	Begin	End	Recombinant Sequence(s)	Minor Parental Sequence(s)	Major Parental Sequence(s)	RDP	GENECONV	Bootscan	Maxchi	Chimaera	SiSscan	3Seq
**1**	9770	1427	OP404087	MT974520.1	OK181760.1<br>AM909717.1<br>AJ972878.1<br>LN832362.1<br>KU987835.1<br>KR296797.1<br>GQ981316.1	1.87E-22	1.74E-109	2.25E-107	6.62E-35	3.29E-36	2.44E-48	5.67E-111
**2-**	120	1428	^JX088636.1	MT974520.1	Unknown<br>(MN207122.1)<br>(GU170807.1)<br>(GU170808.1)<br>(MN508959.1)<br>(MN508960.1)	1.46E-43	8.47E-18	4.63E-34	4.55E-22	6.37E-17	1.52E-29	2.58E-21
**3**	9188	9535	MN508959.1	AJ972878.1<br>OK181760.1<br>AM909717.1<br>LN832362.1<br>KU987835.1<br>KR296797.1	GU170807.1<br>GU170808.1	1.00E-22	2.35E-21	7.21E-23	1.40E-05	1.26E-05	5.58E-05	1.48E-13
**4**	2526	4883	MN508959.1<br>MN207122.1[P]<br>GU170807.1<br>GU170808.1	MT787292.1<br>MK405594.1	MT782116.1<br>OP404087	2.36E-22	NS	1.06E-22	1.01E-14	3.16E-09	1.60E-20	2.51E-10

			MN508960.1								
**5**	2309	2450*	^MN508959.1	Unknown<br>(OK181760.1)<br>(AM909717.1)<br>(AJ972878.1)<br>(LN832362.1)<br>(KU987835.1)<br>(KR296797.1)	GU170808.1<br>GU170807.1	1.95E-16	1.70E-15	1.35E-16	4.00E-05	7.62E-05	7.03E-05	4.12E-09
**6-**	5656	5909	^MN207122.1	GU170807.1	Unknown<br>(GU170808.1)	1.02E-15	6.68E-13	7.45E-16	4.00E-10	2.36E-08	4.30E-13	5.54E-06
**7-**	5736	5894	^GU170808.1	Unknown<br>(MT974520.1)<br>(KC711055.1)<br>(KC711056.1)<br>(MK405594.1)<br>(MT787292.1)	GU170807.1	2.28E-15	6.47E-05	1.31E-15	1.04E-03	8.06E-05	NS	3.06E-08
**8**	8336	8520	^GU170808.1<br>MN207122.1<br>GU170807.1	AJ972878.1<br>OK181760.1<br>AM909717.1<br>LN832362.1<br>KR296797.1	MN508959.1	2.95E-11	3.19E-10	2.33E-11	9.34E-03	4.77E-02	1.97E-02	3.12E-05
**9**	957	1498	MN508959.1	MN508960.1	GU170807.1<br>GU170808.1	1.55E-09	7.52E-03	1.65E-09	5.08E-10	1.52E-09	3.62E-07	3.75E-08
**10**	6854	6924	MN508959.1	AJ972878.1<br>OK181760.1<br>AM909717.1	GU170808.1<br>GU170807.1	2.97E-10	8.17E-10	9.95E-10	NS	NS	NS	3.25E-05

	LN832362.1	
	KU987835.1	
	KR296797.1	
	GQ981316.1	
	OP404087	

- = It is possible that this apparent recombination signal could have been caused by an evolutionary process other than recombination.

* = The actual breakpoint position is undetermined (it was most likely either overprinted by a subsequent recombination event or off the edges of the analysed sequence fragments).

^ = The recombinant sequence may have been misidentified (one of the identified parents might be the recombinant)

Minor Parent = Parent contributing the smaller fraction of sequence.

Major Parent = Parent contributing the larger fraction of sequence.

Unknown = Only one parent and a recombinant need be in the alignment for a recombination event to be detectable. The sequence listed as unknown was used to infer the existance of a missing parental sequence.

NS = No significant P-value was recorded for this recombination event using the particular method in question.
