# Supplementary figures and images for: The Additional 15 nt of 5′ UTR in a Novel Recombinant Isolate of Chilli Veinal Mottle Virus in Solanum nigrum L. Is Crucial for Infection
Source: Viruses. 2023 Jun 23;15(7):1428. doi: 10.3390/v15071428 (PMC10384581; doi:10.3390/v15071428)

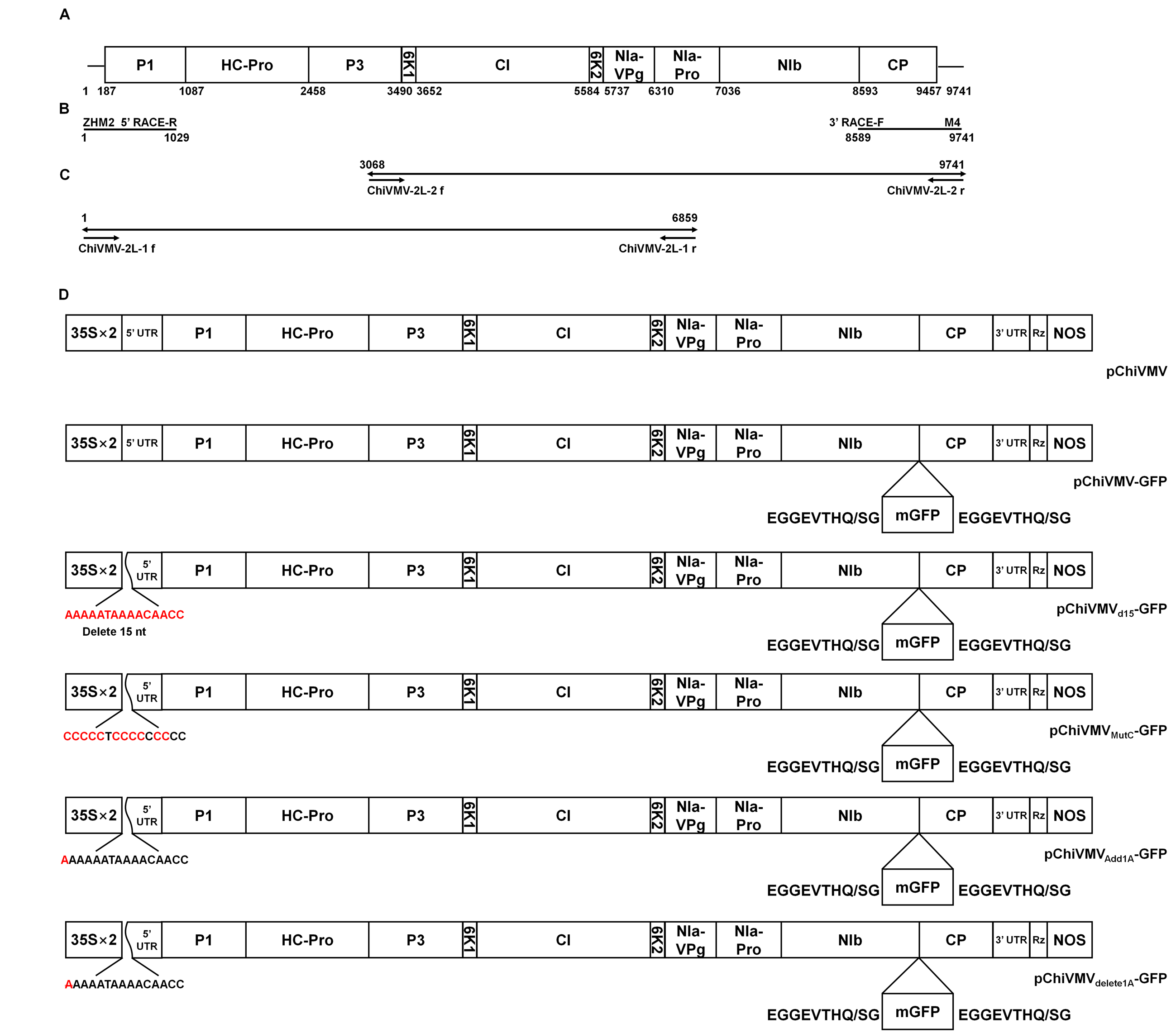

Supplement: Supplementary file 1 [file viruses-15-01428-s001.zip › Figure S1.tif]

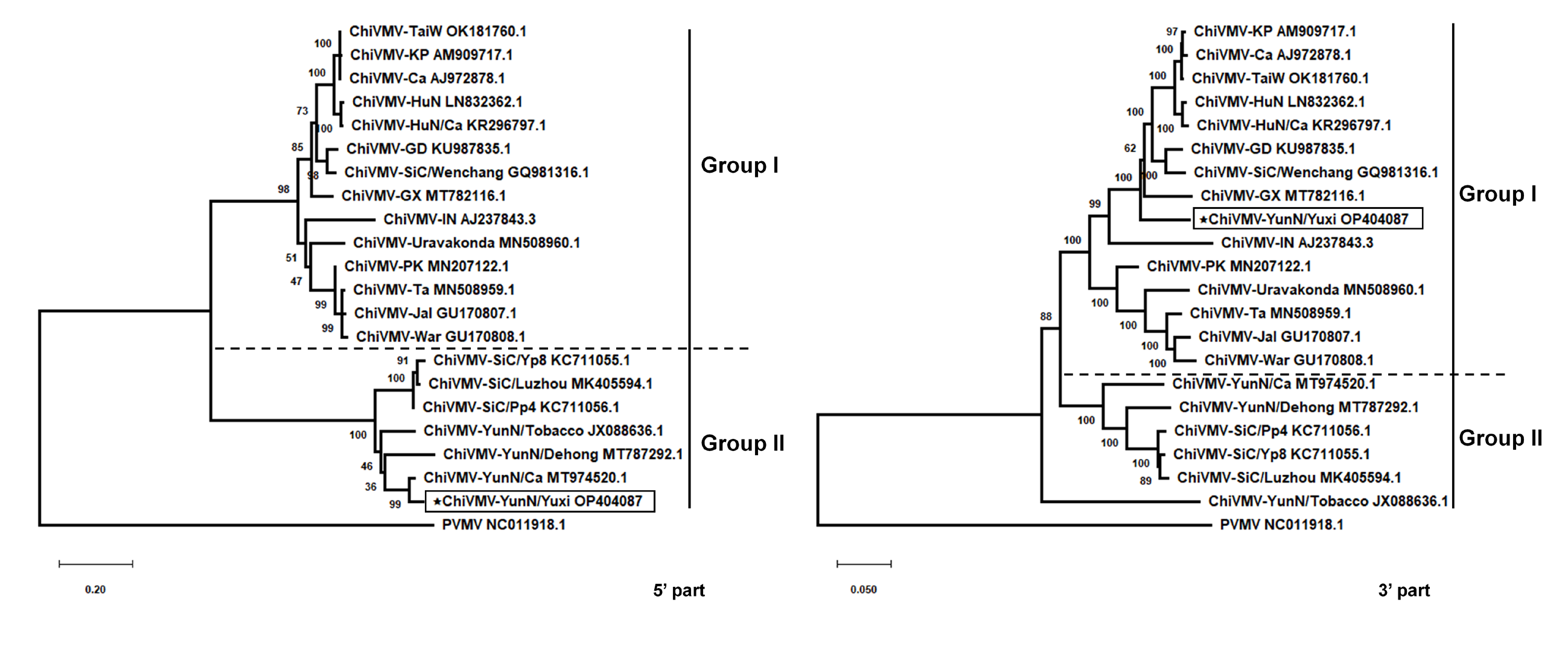

Supplement: Supplementary file 1 [file viruses-15-01428-s001.zip › Figure S2.tif]

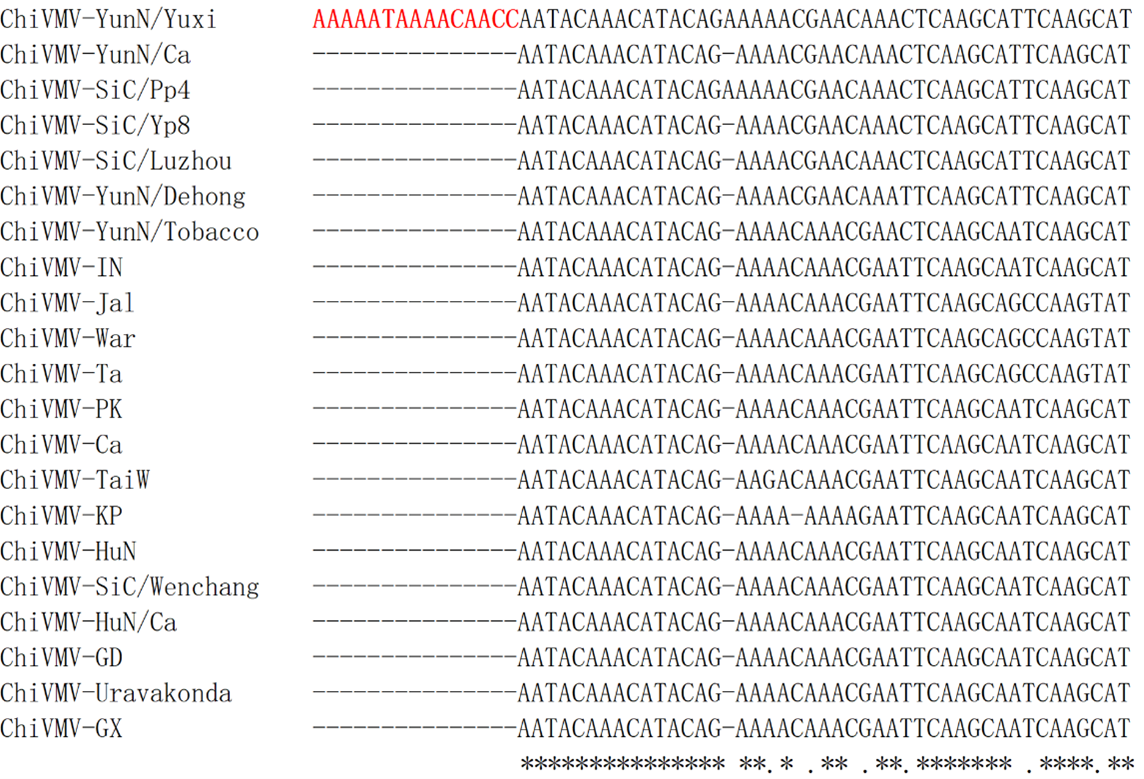

Supplement: Supplementary file 1 [file viruses-15-01428-s001.zip › Figure S3.TIF]

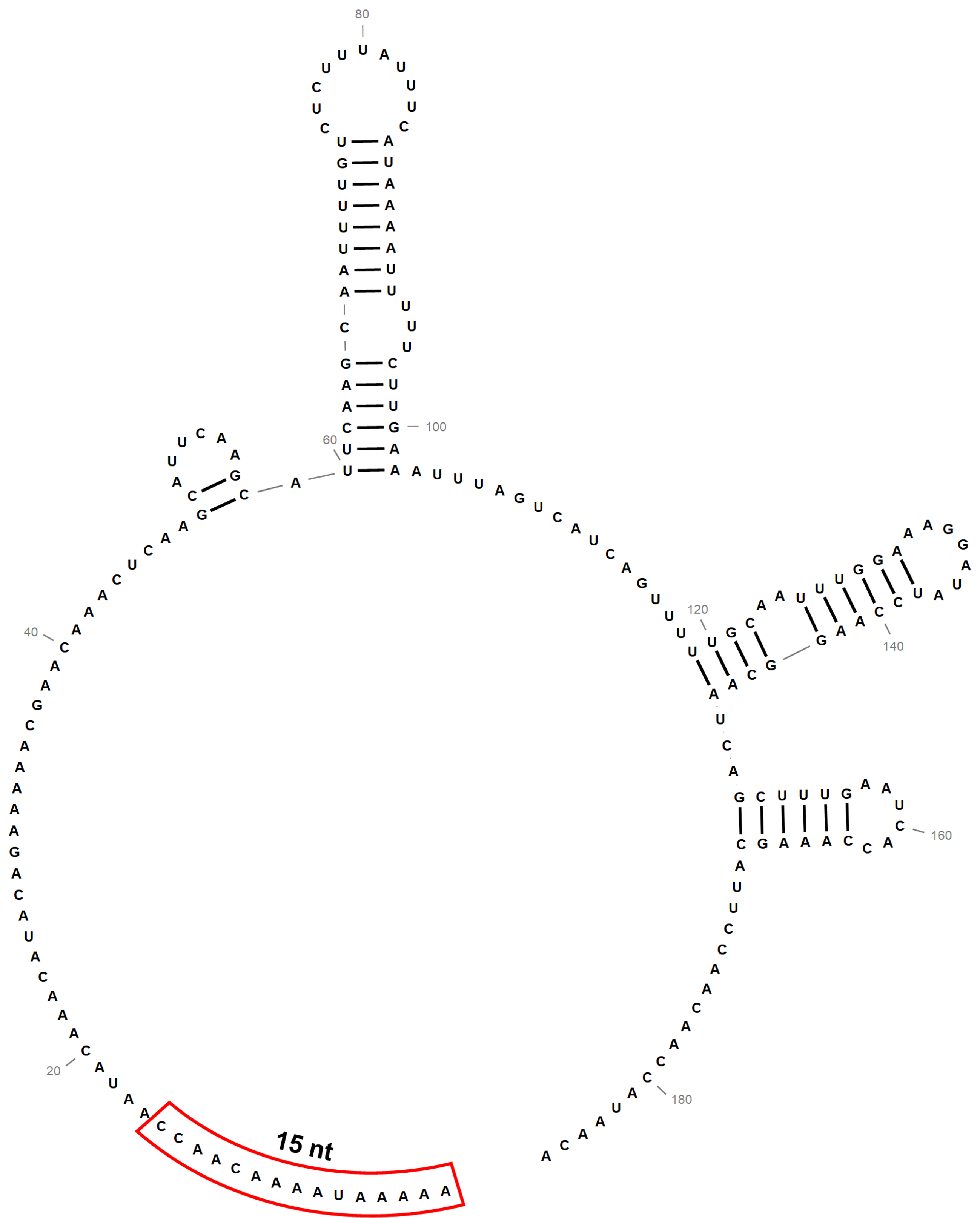

Supplement: Supplementary file 1 [file viruses-15-01428-s001.zip › Figure S4.tif]
